# Regional Expression of Dystrophin Gene Transcripts and Proteins in the Mouse Brain

**DOI:** 10.3390/cells14181441

**Published:** 2025-09-15

**Authors:** Konstantina Tetorou, Artadokht Aghaeipour, Shunyi Ma, Talia Gileadi, Amel Saoudi, Pablo Perdomo Quinteiro, Jorge Aragón, Maaike van Putten, Pietro Spitali, Cecilia Montanez, Cyrille Vaillend, Jennifer E. Morgan, Federica Montanaro, Francesco Muntoni

**Affiliations:** 1 The Dubowitz Neuromuscular Centre, UCL Great Ormond Street Institute of Child Health, London WC1N 1EH, UK; a.dehkaei@ucl.ac.uk (A.A.); shunyi.ma.19@alumni.ucl.ac.uk (S.M.); jennifer.morgan@ucl.ac.uk (J.E.M.);; 2 National Institute for Health Research, UCL Great Ormond Street Institute of Child Health, London WC1N 1EH, UK; 3 Centre National de la Recherche Scientifique (CNRS), Institut des Neurosciences Paris-Saclay, Université Paris-Saclay, 91400 Saclay, Francejaragon@cinvestav.mx (J.A.); cyrille.vaillend@universite-paris-saclay.fr (C.V.); 4 Human Genetics Department, Leiden University Medical Center, 2333 Leiden, The Netherlandsm.van_putten@lumc.nl (M.v.P.);; 5 Departamento de Genética y Biología Molecular, Centro de Investigación y de Estudios Avanzados del Instituto Politécnico Nacional (CINVESTAV), Mexico City 07360, Mexico; cecim@cinvestav.mx

**Keywords:** dystrophin, brain, isoforms, Duchenne muscular dystrophy, mouse models

## Abstract

Duchenne muscular dystrophy (DMD) is a severe neuromuscular disease caused by mutations in the *DMD* gene, leading to muscle degeneration and shortened life expectancy. Beyond motor symptoms, DMD patients frequently exhibit brain co-morbidities, linked to loss of brain-expressed dystrophin isoforms: most frequently Dp427 and Dp140, and occasionally Dp71 and Dp40. DMD mouse models, including *mdx^5cv^* and *mdx52*, replicate key aspects of the human cognitive phenotype and recapitulate the main genotypic categories of brain phenotype. However, the spatio-temporal expression of brain dystrophin in mice remains poorly defined, limiting insights into how its deficiency disrupts brain development and function. We systematically mapped RNA and protein expression of brain dystrophin isoforms (Dp427 variants, Dp140, Dp71, and Dp40) across brain regions and developmental stages in wild-type mice. Dp427 isoforms were differentially expressed in the adult brain, with Dp427c enriched in the cortex, Dp427p1/p2 in the cerebellum, and Dp427m was also detected across specific brain regions. Dp140 was expressed at lower levels than Dp427; Dp71 was the most abundant isoform in adulthood. Dp140 and Dp71 displayed dynamic developmental changes, from E15 to P60, suggesting stage-specific roles. We also analysed *mdx^5cv^* mice lacking Dp427 and *mdx52* mice lacking both Dp427 and Dp140. Both models had minimal Dp427 transcript levels, likely due to the nonsense-mediated decay, and neither expressed Dp427 protein. As expected, *mdx52* mice lacked Dp140, confirming their genotypic relevance to human DMD. Our study provides the first atlas of dystrophin expression in the wild-type mouse brain, aiding understanding of the anatomical basis of behavioural and cognitive comorbidities in DMD.

## 1. Introduction

Duchenne muscular dystrophy (DMD) is the most common muscular dystrophy affecting children, with an incidence between 1 in 3500 and 5000 live male births. DMD is caused by mutations in the *DMD* gene that prevent dystrophin protein expression. Consequently, DMD patients present with early childhood muscle weakness that progresses rapidly, leading to loss of ambulation by early adolescence. The subsequent respiratory and cardiac insufficiency results in a shortened life span.

**Figure 1:**
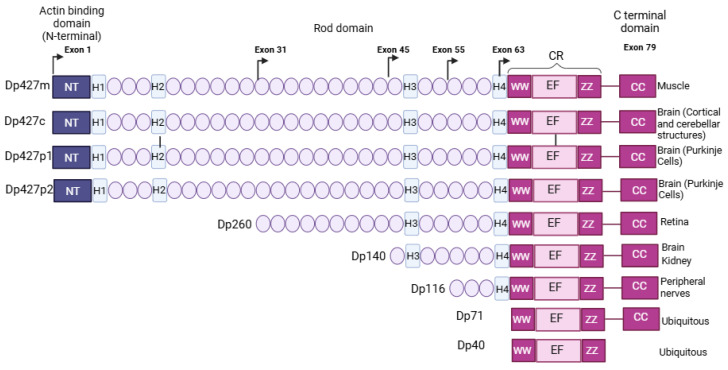
Modular organisation of dystrophin and isoforms. According to the UniProt database (UniProt ID: P11532), all dystrophin isoforms share a common modular structure comprising the cysteine-rich domain (CR). The Dp427 isoforms share an N-terminus (NT) with actin-binding sites from amino acid 1–240; a long central rod domain composed of β-spectrin-like repeats from amino acid 339–1463 and 1468–3040; and proline-rich hinge regions (H1–H4), predicted to form triple-helical coiled-coils. Additional domains are found in the C-terminal region, including the double tryptophan (WW) domain from amino acid 3055–3088 (WW), the zinc-finger (ZZ) domain from amino acid 3308–3364 (helix-loop-helix (EF/ZZ)) within the cysteine-rich region (CR), and the coiled-coil domain (CC) in the C-terminus domain from amino acid 3260–3685. The C-terminal region interacts with specific components of the dystrophin-associated protein complex through these protein domains, such as β-dystroglycan, while actin binds to the rod domain. Notably, the Dp427 isoform and shorter variants differ in the length of their rod domain and the high-affinity actin binding domains located in the NT. Within the shorter isoforms, Dp260 is the only isoform that retains the low-affinity actin binding mediated by the rod domain [10]. This figure was generated using NCBI’s sequence Viewer and protein tools (Version 3.47) [11]. Figure was adapted from Tetorou et al., 2024 [12].

The *DMD* gene is located at chromosome Xp21.2 and produces not only skeletal and cardiac muscle dystrophin protein, but also multiple isoforms mostly expressed in the central nervous system (CNS) (Figure 1) [1]. The gene consists of 79 exons and 7 promoters, each associated with unique first exons [2,3,4]. Among the various isoforms of dystrophin, the full-length isoform, named Dp427 (427 kDa dystrophin protein), can be transcribed through three different tissue-specific promoters, specifically muscle (M), cortical brain regions (C), and cerebellar Purkinje neurons (P) (Figure 1) [1]. In addition to Dp427, shorter dystrophin isoforms (Dp260, Dp140, Dp116, Dp71, and Dp40) are produced via internal independent promoters from and downstream of intron 29, each using specific first exons spliced into exons 30, 45, 56, and 63 to generate the respective isoforms [3,5]. These also undergo alternative splicing, resulting in multiple tissue-specific variants [3].

The promoter of Dp71 [6] also produces the Dp40 variant, the shortest dystrophin product sharing the same 5′-untranslated region (UTR) and the first seven amino acids of Dp71, but with a distinct 3′-UTR originating within the intron 70 sequence [7,8]. Except for Dp40, all Dp71 isoforms share a unique C-terminal region subject to alternative splicing [9].

Dystrophin isoforms also have distinct expression patterns across brain regions and cell types. Previous work in the mouse brain found that Dp427 is mainly located in neurons with a specific role in the localisation and functioning of central γ-Aminobutyric acid type A (GABA_A_) receptors [13]. Dp140 mainly plays a role during early stages of neurodevelopment, and it is also present in the mouse adult brain [14], as well as in the human brain [15,16], with high expression in the embryonic and foetal brain [16]. Notably, Dp140 is present in immature rat-derived oligodendrocytes and nuclear fractions, as indicated in in vitro experiments [17], and is involved in glutamatergic neurotransmission [18]. Dp71 is involved in the functioning of astrocytes, as exhibited in studies on mouse brain and human astrocyte studies using immunofluorescence and immunocytochemistry techniques [19,20].

A variable proportion of DMD individuals (20–50%, depending on the site of the *DMD* mutations) have complex neuropsychiatric and neurocognitive phenotypes, manifesting with specific language disorders, intellectual disability, reading delay, autism spectrum disorder (ASD), attention-deficit/hyperactivity disorder (ADHD), emotional disorders, and/or obsessive compulsive disorders (OCD) [21]. These comorbidities may be, in part, a direct consequence of the lack of brain dystrophins, and the more isoforms that are missing, the more severe the symptoms are [22,23,24,25].

Moreover, a variety of dystrophic animal models have improved our understanding of the aetiology of dystrophinopathy in the brain [26]. DMD mouse models exhibit a range of spontaneous, chemically or transgenically induced mutations in the *Dmd* gene, resulting in a variety of brain dystrophin losses. The most extensively studied Dp427-deficient *mdx* mouse model [27] exhibits enhanced stress reactivity [28,29,30,31], anxiety behaviours [31,32,33], and delays in some learning tasks and long-term memory deficits [32,33]. It is important to note that all strains lacking multiple dystrophin isoforms have a C57BL/6J genetic background, which complicates direct comparisons with the classic *mdx* mouse model, which is on a C57BL/10ScSnJ genetic background [34]. There are various DMD mouse models that hinder the efforts to link the number of missing isoforms to the disease severity. The *mdx^5cv^* mouse, lacking Dp427 [35], is on a C57BL/6J genetic background, thus serving as a possible alternative. The lack of both Dp427 and Dp140 brain isoforms in the *mdx52* mouse model (exon 52 deletion) [36] has been correlated to abnormal social behaviours and diminished glutamatergic transmission, paralleling the ASD-like symptoms of DMD children lacking this isoform [18,36]. At the same time, *mdx52* mice demonstrate higher anxiety in comparison to *mdx* and *mdx^5cv^* mice, suggesting that Dp140 loss exacerbates emotional deficits [32,36]. Indirect factors such as inflammatory mediators have also been involved in some neurobehavioural defects of DMD patients [37].

Our knowledge of the spatio-temporal expression patterns of dystrophin isoforms in the murine brain is, however, still limited, and further research on this could illuminate neurological aspects of DMD. In this study, we therefore aimed to (i) assess dystrophin expression patterns in the wild-type (WT) animals and characterise the spatio-temporal expression patterns of each isoform in the murine brain and (ii) assess what the consequences are of Dp427 absence or concomitant lack of Dp427 and Dp140 on the expression patterns of the remaining isoforms in DMD mouse models using a range of relevant methods (quantitative PCR, single-molecule FISH technologies, capillary, and standard Western blots). To our knowledge, this is the first study detailing the localisation of dystrophin isoforms in the brain of these animal models. We observed that dystrophin isoforms may have distinct spatial and temporal expression patterns. The absence of single or multiple dystrophin isoforms does not affect the expression of the unaffected dystrophin isoforms.

## 2. Materials and Methods

### 2.1. Animals

The *mdx^5cv^* mice were originally generated by chemical mutagenesis [38]. They carry a single A-to-T transversion in exon 10 of the *Dmd* gene, which results in the creation of a new splice donor site. This alteration causes a frameshift with a 53 bp deletion and introduces a stop codon in the mRNA [35]. *Mdx^5cv^* mice (C57BL/6J-B6Ros.Cg-*Dmd^mdx-5Cv^*/J; Jackson Laboratory, Bar Harbor, ME, USA) were purchased from Charles River, London, UK and transferred to University College London.

The *mdx52* mice exhibit exon 52-deleted X chromosome-linked muscular dystrophy. They were generated by Dr. Katsuki Motoya’s group [36] by replacing exon 52 of the *Dmd* gene with a neomycin resistance gene, resulting in the loss of Dp427, Dp260, and Dp140 dystrophin protein expression, while retaining Dp116 protein expression in peripheral nerves and Dp71 and Dp40 protein in the brain and retina [36]. The mouse line was backcrossed with the C57BL/6J strain for more than eight generations and provided by Prof. Sasaoka Toshikuni (Department of Comparative & Experimental Medicine/Brain Research Institute; Niigata University, Niigata, Japan). Breeders were supplied to our lab by Dr. Jun Tanihata and Dr. Shin’ichi Takeda (National Center of Neurology and Psychiatry, Tokyo, Japan).

Both DMD mouse models were generated through the crossbreeding of wild-type (WT; C57BL/6J) males with heterozygous (+/−) females. Genotyping was performed using previously established polymerase chain reaction (PCR) methods on the obtained ear biopsy samples [39]. Mice were housed in individually ventilated cages (IVC, Tecniplast Buguggiate, Italy) in a specific pathogen-free facility on a 12:12 h light/dark cycle with ad libitum access to food (Teklad Global Diet 2018c, Inotiv) and water, while humane endpoints were set to loss of 25% of body weight. All animal experiments and care protocols were approved by the Home Office and carried out according to the UK Animals (Scientific Procedures) Act 1986, following the ARRIVE guidelines.

### 2.2. Tissue Collection

Adult age-matched male C57BL/6J, *mdx^5cv^*, and *mdx52* mice (10–14-week-old, *n* = 5 per mouse line) were euthanised by an increasing dose of CO_2_ for brain collection. After sagittal hemisection, both brain hemispheres were employed for microdissection of cortex, cerebellum, hindbrain, hippocampus, olfactory bulb, and midbrain regions. Brain tissue samples from the right hemisphere were used for RNA quantification. They were submerged in RNAprotect (Cat. No. 76106, Qiagen, Hilden, Germany) and incubated at 4 °C overnight. The RNAprotect solution was then removed, and the tissues were stored at −70 °C until analysis. Samples from the left hemisphere were placed in cryovials and frozen in liquid nitrogen, then stored at −70 °C, after which they were used for protein quantification. Fresh-frozen whole brains (*n* = 5) were also used for in situ experiments. The brains were rinsed in PBS before cutting and then embedded in OCT moulds as follows: 1 × hemisphere sagittal embedded, the other hemisphere was cut coronally into two pieces, one of the cerebellum, and one of the forebrain, before being frozen in a dry ice bath with 100% ethanol.

Foetal brains from male/female C57BL/6J mice (*n* = 2–3 mice per time point) were extracted from the uterine cavity following euthanasia of pregnant females at 15 post-coitum days (E15). Embryos were delicately isolated and deposited in cold phosphate-buffered saline before dissection. The cerebral cortex and cerebellum of these E15 embryonic brains, as well as brains from mice euthanised at birth (P0) and postnatal days P2, P7, P10, P14, P21, and P60, were dissected out, frozen in liquid nitrogen, and stored at −80 °C.

### 2.3. Analysis of Dmd Transcripts via Quantitative Polymerase Chain Reaction

Brain tissues were homogenised using TissueLyser II (Cat. No. 85300, Qiagen) after adding two Tungsten Carbide Beads (Cat. No. 69997, Qiagen) into each tube at 30 cycles/s for 4 min. Samples were then centrifuged at 21,000× *g* for 5 min at room temperature, and RNA was extracted using the RNeasy Mini kit (Cat. No. 74104, Qiagen). The yield and purity of the samples were then measured using a NanoDrop 2000 spectrophotometer (Thermo Fisher Scientific, Waltham, MA, USA) at 260/280 nm, and samples were stored at −70 °C until further use. The RNA samples were reverse-transcribed using the High-Capacity RNA to cDNA kit (Cat. No. 4387406, Thermo Fisher Scientific). Following the protocol, the cDNA was made from 1 µg of RNA in each sample. An additional step of DNase treatment (Cat. No. 18068015, Invitrogen, Waltham, MA, USA) was performed to minimise the genomic DNA (gDNA) contamination in the cDNA samples since the probe for Dp140 and Dp40 recognises gDNA.

Amplification was performed using Taqman fast advanced master mix (Cat. No. 4444557, Thermo Fisher Scientific) and custom-designed Taqman probes (Table 1; Integrated DNA technology) per manufacturer instructions on a StepOne real-time PCR detection system (Thermo Fisher Scientific, Horsham, UK). The Taqman assays were designed using Primer Express software (v3.0.1) and validated by examining (i) the predicted specificity—the alignment of the primer sequences on BLAST (v2.16.0) to confirm the specificity of the assay; (ii) PCR efficiency using dilution series- Ct values were plotted against cDNA concentration, and the efficiency of the PCR reaction was calculated from the slope using the formula E = 100 × (−1 + 10(−1/slope)); (iii) PCR efficiency using amplification curves—LinRegPCR software (v2021.2) calculates PCR efficiency directly from qPCR amplification curves. Mean PCR efficiencies were calculated across multiple wells to better estimate the efficiency of each assay. Relative gene expression was calculated using the 2^−∆∆Ct^ method, and the samples were analysed in triplicate. Multiple housekeeping genes were tested (*Ube2d2a*, *Rpl13*, and *Cyc1*), and normalisation was performed to the *Ube2d2a* gene (Assay ID: Mm.PT.58.12995636, Integrated DNA Technology, Coralville, IA, USA) using LinRegPCR software [40], as it was expressed at the same levels across all brain regions tested. The samples of direct comparisons were run on the same plate.

### 2.4. Protein Quantification via Simple Capillary Western Blot (WES)

The protein samples of the adult mice were prepared on ice for extraction after adding 50 μL of a solution made of Protease and Phosphatase Inhibitor Mini Tablets (Cat. No. A32959, Pierce) to 950 μL of T-PER tissue protein extraction reagent (Cat. No. 78510, Thermo Scientific) for each mg of tissue. Tissues were then homogenised using a Fisherbrand 850 Homogenizer (Cat. No. 15505819, Loughborough, UK) for 20 s at 10,000 rpm and placed in an ultrasonic ice bath (Branson 2510-DTH Ultrasonic Cleaner, Branson Ultrasonics, Brookfield, CT, USA) for 5 min. The homogenates were then centrifuged for 15 min at 14,000× *g* at 4 °C; the resulting supernatant was then centrifuged again at 14,000× *g* at 4 °C for 5 min and kept at −70 °C.

Simple capillary Western blot (WES, Bio-techne, Minneapolis, MN, USA) was performed to quantify dystrophin protein in C57BL/6J, *mdx^5cv^* and *mdx52* mouse brain samples. Protein extracted from the samples was quantified using RC DC Protein Assay Reagents Package (Cat. No. 5000120, Bio-Rad Laboratories, Hercules, CA, USA). In total, 2 μg of protein for each sample was prepared for WES analysis following protocols for the 66–440 kDa separation module (Cat. No. SM-W005, Bio-techne) and the total protein detection module (Cat. No. DM-TP01, Bio-techne). A rabbit monoclonal dystrophin antibody (Cat No. Ab154168, 1:1500, Abcam, Cambridge, UK) that recognises all dystrophin isoforms with exons 78 and 79 was used to quantify protein expression using a secondary anti-rabbit horseradish peroxidase (HRP)-conjugated antibody (Cat. No. 042-206, Bio-techne). A chemiluminescent substrate was introduced, reacting with HRP secondary antibody to produce light, followed by capturing of light’s emission using an integrated CCD camera. A semi-quantification method was used to detect Dp427, Dp140 and Dp71 signal after normalisation to the total protein detection signal of each sample.

### 2.5. Protein Quantification via Western Blot

Protein extracts were obtained from embryonic and postnatal mouse brain structures (cortex, cerebellum) treated with RIPA lysis and extraction buffers complemented with SDS (5% final). Total protein concentration was determined with the BCA Protein Assay Kit (Cat. No. 23225, Thermo Fisher Scientific). Samples were denatured at 100 °C for 3 min, and 25 μg of protein was loaded (20 µL per well) onto NuPAGE 3–8% Tris-Acetate Protein gels (Invitrogen).

A 105 min electrophoresis at 100 V allowed us to detect Dp140 and Dp71 on the same blot. Proteins were then transferred to a nitrocellulose membrane for 16 h at 100 mA in a transfer buffer solution bath at 4 °C. The nitrocellulose membrane was blocked with 5% milk in PBS Tween 0.1%. Dystrophin protein was detected by probing the membrane (3 h at room temperature) with primary Rabbit monoclonal antibody (Abcam Cat No. Ab154168) targeting the C-terminus (1:1000), and vinculin was detected as an internal control with the mouse monoclonal hVin-1 primary antibody (1:10,000) (Sigma-Aldrich, Saint-Louis, MO, USA), followed by 3 washes of 10 min in PBS tween 0.1% and incubation (1 h at room temperature) with a corresponding secondary antibody (IRDye 800CW Goat anti-mouse IgG 1:20,000 and IRDye 700CW Goat anti-rabbit IgG 1:20,000). Bands were visualised using the Odyssey CLx system (Li-Cor, Homburg, Germany). Quantification was performed using Empiria Studio software (v3.3) (Li-Cor, Bad Homburg, Germany) and normalised to the internal control (vinculin).

### 2.6. BaseScope Analysis of Dmd mRNA

The BaseScope Duplex assay (Cat. No. 323900, ACDbio, Newark, CA, USA) was used to detect the *Dmd* gene via in situ hybridisation (ISH). Fresh frozen tissue sections were fixed in cold, neutral-buffered formalin and subsequently dehydrated in ethanol baths of increasing concentrations (50%, 70%, and 100%). The assay procedure included hydrogen peroxide treatment, protease treatment, and RNA ISH steps, all performed using the BaseScope Duplex reagent kit (Cat. No. 323800, ACDbio) following the manufacturer’s protocol.

**Figure 2:**
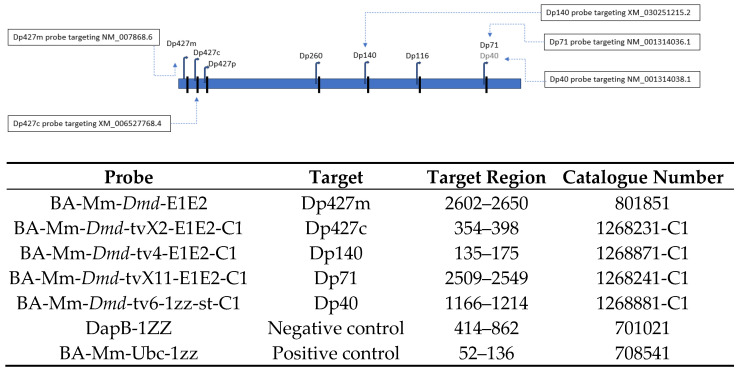
Design and details of BaseScope probes. The picture shows the position of BaseScope probes along the *Dmd* gene. National Center for Biotechnology Information (NCBI) reference sequences of mRNA: NM_007868.6, dystrophin protein 1, XM_006527768.4, dystrophin isoform X2, XM_030251215.2 dystrophin isoform X11, NM_001314036.1 dystrophin isoform Dp71, NM_001314038.1, dystrophin isoform Dp40.

The BaseScope probe design strategy relies on ZZ probes, where each probe is made of two independent hybridisation elements that must bind in tandem to the target sequence to produce a detectable signal. Depending on the target RNA, one to three double Z probe pairs were designed to ensure specific hybridisation to the intended mRNA molecule. All probes consisted of one pair of double Z (ZZ); probes BA-Mm-*Dmd*-E1E2 (Dp427m), BA-Mm-*Dmd*-tvX2-E1E2-C1 (Dp427c), BA-Mm-*Dmd*-tv4-E1E2-C2 (Dp140), BA-Mm-*Dmd*-tv4-E1E2-C1 (Dp71), BA-Mm-*Dmd*-tv6-1zz-st-C1 (Dp40) (Figure 2), and tests were performed according to the manufacturer’s protocol (Advanced Cell Diagnostics Inc., Newark, CA, USA, Cat. No.323900-USM). For each specific brain sample, obtained from C57BL/6J, *mdx^5cv^*, and *mdx52* mice, sections were hybridised with probes targeting the prioritised *Dmd* isoforms. The DapB (4-hydroxy-tetrahydrodipicolinate reductase)-negative control probe was used to establish non-specific labelling, and the BA-Mm-Ubc-1zz—*Mus musculus* ubiquitin C (Ubc) mRNA-positive control probe was used to confirm preservation of RNA in fresh frozen samples. Tissue sections were then counterstained with haematoxylin Gill’s I (GHS132-1L, Sigma) diluted to 50% in water and applied for 30 s, followed by staining with ammonium hydroxide (Cat. No. 205840025, Acros Organics, Geel, Belgium) diluted to 0.02% in water, also for 30 s. Slides were subsequently dried at 60 °C for 10 min and mounted using Vectamount mounting medium (Cat. No. 321584, ACDbio). High-resolution images of the stained tissues were acquired using the NanoZoomer S20 scanner (Hamamatsu Photonics, Shizuoka, Japan) at 40× magnification. Image analysis was performed with NDP.view2 software (v2.9.29) (Hamamatsu Photonics). The Allen mouse brain atlas was used to define regions of interest:—paracentral gyrus, cingulate gyrus, corpus callosum, amygdala, hippocampus, substantia nigra, and cerebellar structures (dentate nucleus and vermis)—and to manually annotate them on the imaged sections. For each annotated region, a set of images (from three randomly selected fields of view) was exported. Then, signal quantification was performed manually in sagittal sections of full brain sections from five mice per group by one individual blinded assessor. Punctuated red dots indicate dystrophin signals, with each dot corresponding to a single mRNA molecule. Only signals that were clearly distinguishable from the background and matched the expected size and intensity (~1–2 μm in diameter) were counted. Background staining and diffuse colouration were excluded from analysis. Thresholding was performed visually and applied consistently across all samples. The number of *Dmd* RNA dots was then normalised per mm^2^ of each analysed image.

### 2.7. Statistical Analysis

The statistical analysis was performed using GraphPad Prism v9.0. Each data set was checked for equality of variance (F test) and for normality with D’Agostino–Pearson normality, Shapiro–Wilk, and Kolmogorov–Smirnov tests. Further, a Kruskal–Wallis test with Dunn’s post hoc analysis was carried out when data were not normally distributed or when variances were not comparable, to assess evidence for sub-regional differences. The *p*-values were corrected with Benjamini–Hochberg multiple hypothesis testing. In the case of normally distributed data, a two-way ANOVA with Bonferroni’s post hoc correction was performed. The results were considered significant when a *p*-value < 0.05 was obtained. The results are presented as mean ± standard error of mean (SEM) of each data set.

## 3. Results

### 3.1. Regional Expression of Dystrophin mRNAs in the Adult Mouse Brain

**Figure 3:**
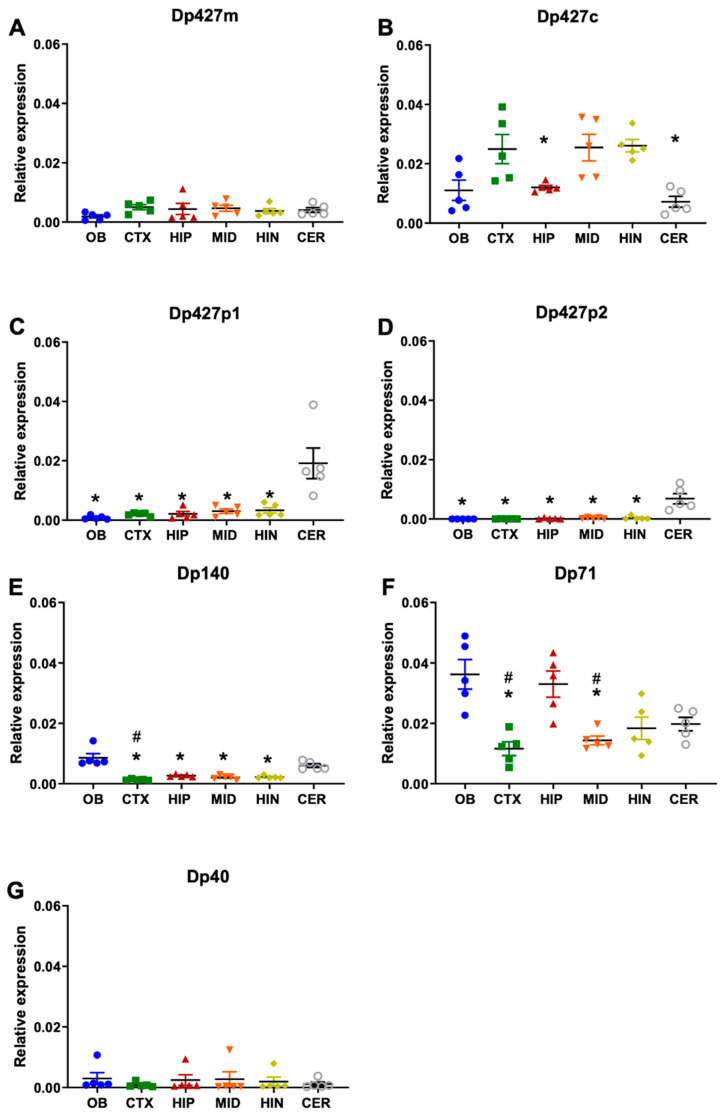
Expression of different dystrophin isoforms across selected C57BL/6J mouse brain regions. (**A**–**D**) Dp427m RNA levels were between 20 and 60% lower compared to Dp427c and did not differ in expression between brain regions. Dp427c is the *Dmd* isoform with higher levels of RNA in cortex and mid- and hindbrain, while Dp427p1 and p2 had higher levels in cerebellum compared to all other brain areas. (**E**–**G**) Dp140 had higher levels in the olfactory bulb, Dp71 had higher levels in hippocampus and olfactory regions, and no sub-regional differences were observed for Dp40. Data were analysed via Kruskal–Wallis test with Dunn’s post hoc multiple comparisons and are presented as mean ± SEM; The results are presented relative to reference gene Ube2d2a as mean ± SEM. Each brain sample was assessed in triplicate. * *p* < 0.05 compared to cortex (**B**), to cerebellum (**C**,**D**), to olfactory bulb (**E**,**F**). # *p* < 0.05 compared to cerebellum (**E**), to hippocampus (**F**). OB: olfactory bulb, CTX: cortex, HIP: hippocampus, MID: midbrain, HIN: hindbrain, CER: cerebellum, (11-week-old male mice, *n* = 5).

RNA transcript levels of the full-length isoforms Dp427m, c, p1,p2 and shorter isoforms (Dp140, Dp71, and Dp40) were quantified via qPCR in the following micro-dissected brain areas of C57BL/6 mice: olfactory bulb, cortex, hippocampus, midbrain, hindbrain, and cerebellum (11-week-old mice, *n* = 5). It is important to note that several housekeeping genes were tested to find one that was expressed at similar levels across all mouse brain regions. RNA transcripts of the muscle isoform of Dp427 (Dp427m) were present at low levels throughout the adult mouse brain with no significant sub-regional differences (Figure 3A). Dp427c (cortical isoform) RNA levels were significantly higher in the cortex compared to hippocampus and cerebellum (Figure 3B, *p =* 0.02, *p* = 0.0016, respectively). Transcripts of Dp427 p1 and p2 isoforms expressed in Purkinje neurons were more expressed in the cerebellum compared to all brain regions analysed (Figure 3C,D, *p =* 0.03, *p =* 0.04, respectively). The analysis of the shorter isoforms showed that Dp140 RNA levels were higher in the olfactory bulb compared to the cortex (*p =* 0.0001), hippocampus (*p* = 0.02), midbrain (*p* = 0.01) and hindbrain (*p* = 0.02), and cerebellum compared to the cortex (Figure 3E, *p* = 0.001). Dp71 levels were highest in the olfactory bulb and hippocampus compared to the cortex (*p* = 0.002, *p* = 0.006, respectively) and midbrain (*p* = 0.01, *p* = 0.02, respectively) (Figure 3F). Lastly, RNA transcript levels of Dp40 showed no sub-regional differences.

**Figure 4:**
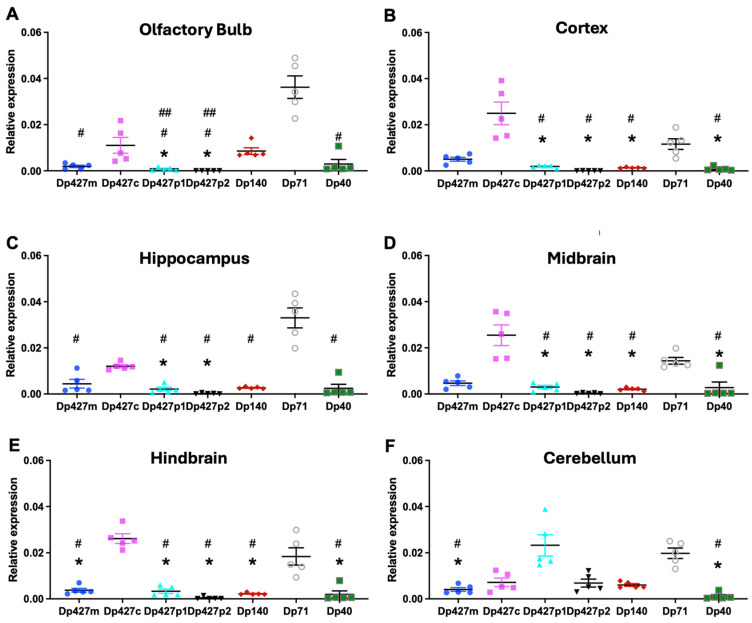
Regional brain differences in transcript levels of dystrophin isoforms in C57BL/6J mice quantified with qPCR. (**A**–**F**) Dp427c is the isoform with highest expression in cortex, midbrain, and hindbrain; Dp427p1 is the isoform with highest expression in cerebellum; and Dp71 is the isoform with highest expression in olfactory bulb and hippocampus. Data were analysed via Kruskal–Wallis test with Dunn’s post hoc multiple comparisons and are presented as mean ± SEM. The results are presented relative to reference gene Ube2d2a ± SEM, *n* = 5. Each brain sample was assessed in triplicate. * *p* < 0.05 compared to Dp427c (**A**–**E**), to Dp427p1 (**F**) # *p* < 0.05 compared to Dp71 (**A**–**F**), ## *p* < 0.05 compared to Dp140.

We also compared the expression of all dystrophin’s RNA isoforms in each dissected brain structure (Figure 4). In more detail, in the olfactory bulb, Dp71 RNA transcript levels were highest and significantly different from Dp427m, p1, p2, and Dp40, while Dp427c levels were significantly higher compared to Dp427p1 and p2 (Figure 4A, *p* = 0.02). In the same brain area, Dp140 levels were higher compared to Dp427p1, p2 (Figure 4A, *p* = 0.04). In the cortex, Dp427c was the dystrophin isoform with the highest RNA transcript levels, followed by Dp71, with a significant difference from Dp427p1, p2, Dp140, and Dp40 (Figure 4B, *p* = 0.001, *p* = 0.02, respectively). In the hippocampus, Dp71 RNA levels were significantly higher compared to all dystrophin isoforms analysed (*p* = 0.001), followed by Dp427c compared to Dp427p1 and Dp427p2 (Figure 4C, *p* = 0.03 and *p* = 0.04, respectively). The same pattern of RNA transcripts as in the cortex was observed in the midbrain and hindbrain (Figure 4D,E, *p* < 0.05). It must be noted that in the hindbrain, Dp427m RNA levels were significantly lower compared to Dp427c and Dp71 (Figure 4E, *p* = 0.04). Lastly, in the cerebellum, Dp427p1 was majorly expressed along with Dp71, and both RNA levels were higher compared to Dp427m and Dp40 (Figure 4F, *p* = 0.001, *p* = 0.02, respectively).

**Figure 5:**
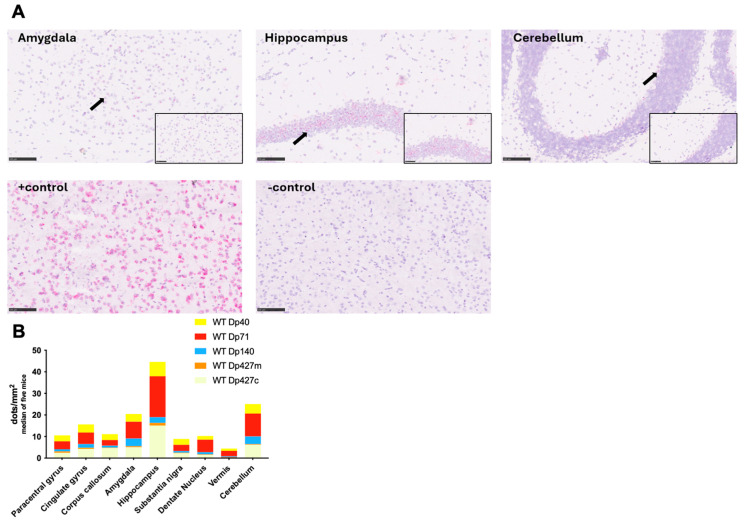
*Dmd* transcript levels across C57BL/6J mouse brains representing the differences between isoforms in different brain areas with BaseScope analysis. (**A**) Representative images of BaseScope hybridisation using Dp71 probe in amygdala, hippocampus, and cerebellum of the C57BL/6J mouse brain. Each red dot corresponds to a single *Dmd* RNA molecule. Black arrows are pointing to red dots representing the RNA transcript. Scale bars = 50 μm. (**B)** The graph shows the number of *Dmd* RNA dots normalised per mm^2^ of the analysed image. Overall, *Dmd* isoforms were transcribed in all brain areas. The results are presented as median of *n* = 5. Note: all dystrophin isoforms have higher RNA levels in hippocampus compared to paracentral gyrus (fold change = 19), corpus callosum (fold change = 10), substantia nigra (Fold change = 21), vermis (fold change = 7), and dentate nucleus (fold change = 10) (*p* < 0.05). Dp427m RNA levels were very low, while Dp140 and Dp40 were expressed at lower levels compared to Dp427c and Dp71 (*p* < 0.05). In amygdala, Dp71 levels were significantly higher compared to Dp427m, Dp140, and Dp40 (*p* < 0.05).

To test in more detail the sub-regional differences of dystrophin’s isoforms transcripts levels, BaseScope analysis was performed on control adult male C57BL/6J samples. The brain regions analysed were the paracentral gyrus, cingulate gyrus, corpus callosum, amygdala, hippocampus, substantia nigra, and cerebellar structures (dentate nucleus and vermis). Dystrophin isoforms were expressed in all mouse brain areas studied with sub-regional differences (Figure 5). Specifically, the hippocampus was the brain area with the highest expression of *Dmd* RNA of each isoform in control C57BL/6J mice, and the expression of Dp427c and Dp71 was most pronounced in the majority of analysed brain areas. In more detail, all dystrophin isoforms had higher RNA levels, as judged by the RNA dots detected by BaseScope in the hippocampus compared to the paracentral gyrus, corpus callosum, substantia nigra, vermis, and dentate nucleus (Figure 5B, *p* < 0.001). Dp427c transcript was widely expressed in all neocortical areas, such as in the cingulate gyrus, and had frequent expression in the CA1-CA2 areas of the hippocampus, with lower expression in CA3, hilum/CA4, and granule cells in the dentate gyrus. Dp427m RNA transcripts showed very low levels but were present in the adult C57BL/6J mice, as quantified by the number of dots detected, while Dp140 and Dp40 were expressed at lower levels compared to Dp427c and Dp71, with no particular differences in the levels of expression in cortex and hippocampus. The number of dots detected for Dp140 was significantly higher in the cerebellum and amygdala compared to the paracentral gyrus, corpus callosum, substantia nigra, vermis, and dentate nucleus (Figure 5B, *p* = 0.009). In the amygdala, Dp71 levels were significantly higher compared to Dp427m, Dp140, and Dp40 (Figure 5B, *p* = 0.003). Dp71 expression in the cortex was similar to Dp427c, although in the hippocampus, Dp71 was more highly expressed in the granule neurons of the dentate gyrus and less in CA1-CA3 and hilum/CA4 areas. In the cerebellum, the expression of all *Dmd* transcripts was more frequent in the Purkinje neurons and reduced in the granular layer.

### 3.2. Regional Expression of Dmd mRNAs in Adult mdx^5cv^ and mdx52 Mice

**Figure 6:**
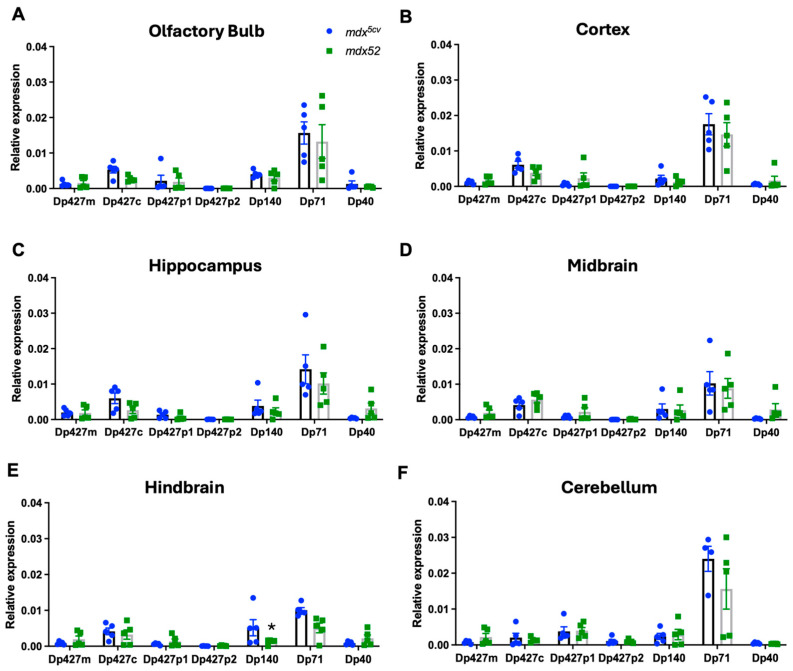
*Dmd* transcript levels in the brain of the *mdx^5cv^* and *mdx52* mice quantified with qPCR. (**A**–**F**) *Dmd* transcript levels for Dp427m, Dp427c, Dp427p1, and Dp427p2 full-length isoforms and Dp140, Dp71, and Dp40 shorter isoforms in olfactory bulb, cortex, hippocampus, midbrain, hindbrain, and cerebellum of *mdx^5cv^* and *mdx52* DMD mouse models. Data were analysed via Kruskal–Wallis test with Dunn’s post hoc multiple comparisons and are presented as mean ± SEM. The results are presented relative to reference gene Ube2d2a, *n* = 5. Each brain sample was assessed in triplicate, * *p* < 0.05.

**Figure 7:**
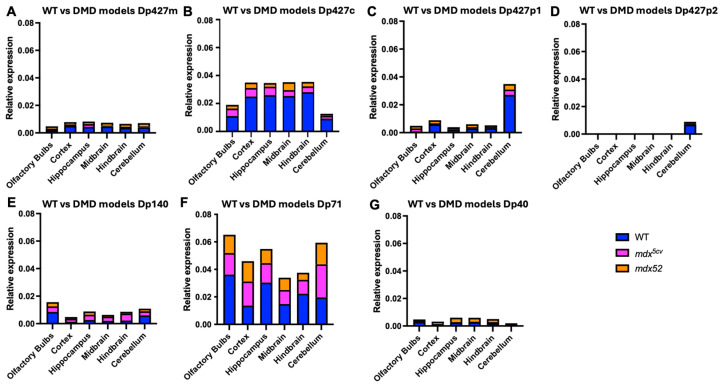
*Dmd* transcript levels in C57BL/6J mice compared to DMD models assessed with RT-qPCR. (**A**–**C**) Dp427m, Dp427c, and Dp42p1 were still present in *mdx^5cv^* and *mdx52* mice in lower levels. (**D**) Dp427p2 levels were present in the cerebellum of C57BL/6J. (**E**) The levels of Dp140 were lower in *mdx^5cv^* and *mdx52* mice compared to C57BL/6J. (**F**,**G**) Dp71 and Dp40 levels were unchanged in *mdx^5cv^* and *mdx52* models. The graphs show the number of *Dmd* RNA dots normalised per mm^2^ of analysed images of *n* = 5 mice per strain.

We also aimed to investigate the levels of dystrophin isoform gene expression in DMD models, i.e., *mdx^5cv^* lacking Dp427 and *mdx52* lacking both Dp427 and Dp140. To achieve this, we analysed the levels of all dystrophin isoforms with qPCR and BaseScope. The expression of the full-length isoforms was lower in both DMD models compared to the C57BL/6J samples due to their mutation affecting the expression of these isoforms, as well as Dp140 being affected only in the *mdx52* model. In the hindbrain, Dp140 was significantly lower in *mdx52* mice compared to *mdx^5cv^* mice (*p* = 0.0032). The mutations present in those two DMD mouse models did not affect the expression patterns of the Dp71 and Dp40 isoforms (Figure 6). A comparison between C57BL/6J and DMD models of qPCR results is presented in Figure 7.

**Figure 8:**
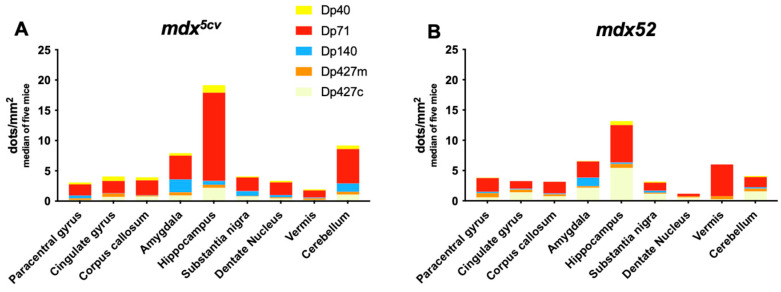
*Dmd* transcript levels in mouse brain of *mdx^5cv^* and *mdx52* mice assessed with BaseScope. (**A**) In *mdx^5cv^* mice, which lack Dp427, Dp71 was expressed at highest levels in all brain regions assessed, while very low to no expression of Dp427c and Dp427m was observed. (**B**) In the *mdx52* model, which lacks Dp427 and Dp140, Dp71 is less expressed than in the *mdx^5cv^* model. The graphs show the number of *Dmd* RNA dots normalised per mm^2^ of analysed images of *n* = 5 mice per strain.

**Figure 9:**
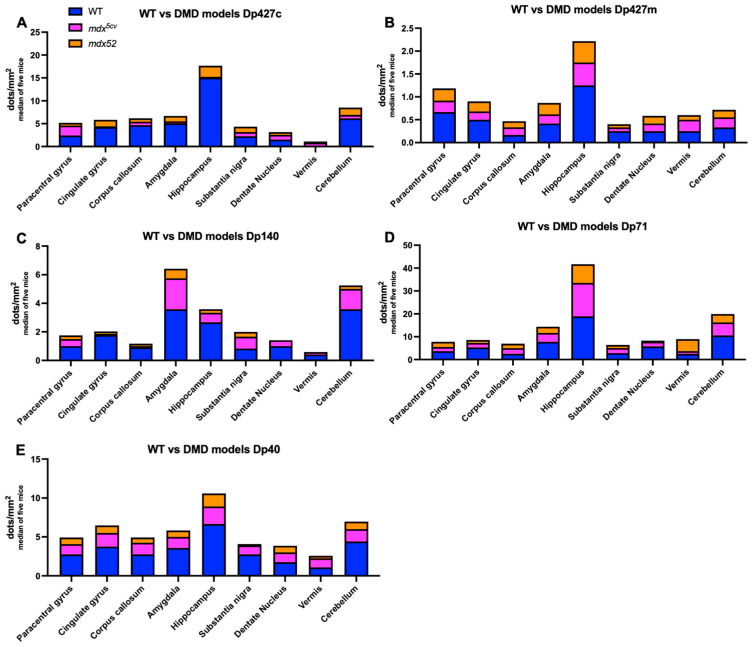
*Dmd* transcript levels in C57BL/6J mice compared to DMD models assessed with BaseScope. (**A**,**B**) Dp427c and Dp427m were still present in *mdx^5cv^* and *mdx52* mice in lower levels. (**C**) The levels of Dp140 were lower in *mdx^5cv^* and *mdx52* mice compared to C57BL/6J. (**D**,**E**) Dp71 and Dp40 levels were unchanged in *mdx^5cv^* and *mdx52* models. The graphs show the number of *Dmd* RNA dots normalised per mm^2^ of analysed images of *n* = 5 mice per strain.

BaseScope analysis demonstrated that in the Dp427-deficient *mdx^5cv^* mice, Dp71 was expressed at the highest levels in all brain regions assessed, similarly to C57BL/6J mice. However, very low levels to no expression of Dp427c and Dp427m was observed, which is likely the result of mRNA nonsense-mediated decay, coupled with the previously reported transcript imbalance observed for the *Dmd* mRNA [41]. Finally, we found that there was a tendency for Dp71 levels to be reduced in the *mdx52* model (Figure 8) (*p* = 0.07). In Figure 9, a comparison of *Dmd* transcripts for each isoform is presented between C57BL/6J and DMD models.

### 3.3. Regional Expression of Dystrophin Proteins in the Mouse Brain

**Figure 10:**
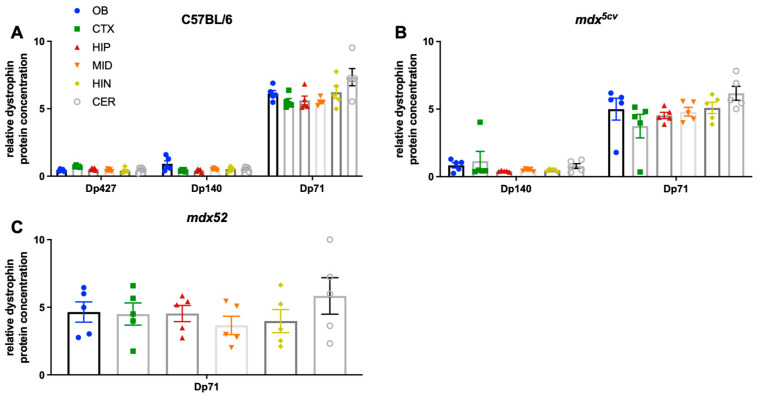
Dystrophin isoforms protein expression in the adult C57BL/6J, *mdx^5cv^,* and *mdx52* mouse brain assessed via simple capillary Western. (**A**) Dp71 was highly expressed compared to Dp140 and Dp427 in all assessed brain regions of C57BL/6J mice (*n* = 5), with no sub-regional differences. Total protein was quantified per individual sample using the total protein detection module. (**B**,**C**) There was no Dp427 expression in *mdx^5cv^* mice (*n* = 5) lacking Dp427, and no Dp427 and Dp140 expression in *mdx52* mice (*n* = 5) lacking both Dp427 and Dp140, while Dp71 maintained high levels in both DMD models. Dystrophin protein levels were normalised to total protein assay ± SEM. OB: olfactory bulb, CTX: cortex, HIP: hippocampus, MID: midbrain, HIN: hindbrain, CER: cerebellum.

In this study, we also aimed to analyse the regional expression of dystrophin proteins in C57BL/6J mice. Using the WES method, we found that Dp427, Dp140, and Dp71 protein levels present no sub-regional differences in C57BL/6J mice (Figure 10). Dp71 was the most highly expressed protein in all the brain regions assessed (olfactory bulb, cortex, hippocampus, midbrain, hindbrain, and cerebellum) (Figure 10). This is in contrast to the RNA levels detected with qPCR, where Dp427c is highly expressed in most of the brain regions analysed, and Dp427p is more highly expressed in the cerebellum compared to Dp71. It must be noted that, due to a lack of specific antibodies for each Dp427 isoform, the results present the total levels of Dp427 in the C57BL/6J mouse brain, i.e., the cumulative levels of Dp427c, m and p. We also assessed the protein levels of dystrophin isoforms in the DMD mouse models. There was no Dp427 protein in either DMD mouse model (*mdx^5cv^* and *mdx52*), while Dp140 was only deficient in *mdx52* mice. Dp140 levels were unchanged in the *mdx^5cv^* mouse, as expected (Figure 10). Dp71 levels were not affected in the two DMD mouse models (Figure 10).

### 3.4. Expression Levels of Dystrophin Proteins During Development of C57BL/6J Mice

**Figure 11:**
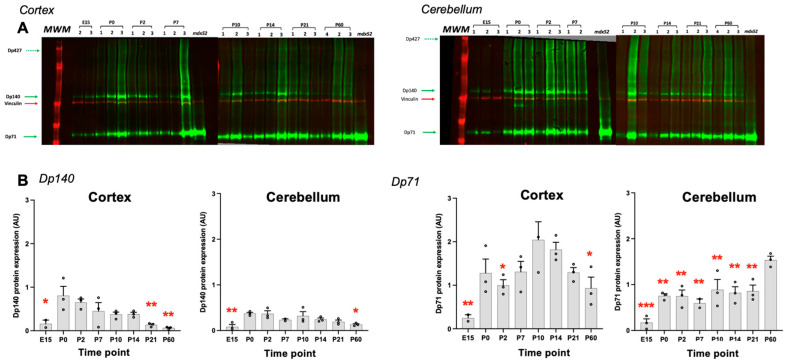
Dp140 and Dp71 protein levels during development of the mouse brain. (**A**) Western blot detection of Dp140 and Dp71 in the cortex and cerebellum of WT embryos (E15) and at postnatal ages (P0 to P60). Note the absence of immunoreactive signal at 140 kDa in *mdx52* mice, confirming lack of Dp140 in this model, and validating that the 140 kDa band corresponds to Dp140 in WT samples. MWM: molecular weight marker. Expression was normalised to Vinculin (red arrow). Dp427 showed very low expression in this experimental condition and was not quantified. (**B**) Quantifications of Dp140 and Dp71 protein levels as indicated. *n* = 2–3 mice per time point, as shown in the Western blots in (**A**). * *p* < 0.05, ** *p* < 0.01, *** *p* < 0.001, P0 compared to other ages for Dp140 (both cortex and cerebellum), P10 compared to other ages for Dp71 in cortex, or P60 compared to other ages for Dp71 in cerebellum, one-way ANOVAs followed by Holm–Sidak multiple comparisons.

To investigate the developmental expression of Dp140 and Dp71 proteins, a Western blot analysis was performed from embryonic (E15) to postnatal and adult (P60) ages of wild-type mice using a pan-dystrophin monoclonal rabbit antibody directed against the common C-terminus of all dystrophins (Figure 11A). In both cortex and cerebellum, Dp140 expression levels drastically increased between E15 and P0, after which they steadily declined with age during the postnatal period, a critical period of brain plasticity [42], with maximal levels at birth (P0). Dp71 expression was also lower at E15 compared to the postnatal period in both structures and showed higher expression from P0 to P14 in the cortex and at P60 in the cerebellum (Figure 11B). Dp427 protein was not quantifiable due to its very low expression in this experimental condition.

## 4. Discussion

Our study provides a comprehensive analysis of dystrophin isoform expression at the RNA and protein level in the brain of control C57BL/6J mice and two DMD mouse models, *mdx^5cv^* and *mdx52,* that recapitulate dystrophin isoform involvement of about 90% of all DMD boys. This research presents several novel findings that contribute to our understanding of the temporal and spatial expression of dystrophin isoforms in the mouse brain and their potential roles in neurodevelopment and neuropathophysiology within the context of DMD.

One of the most novel contributions of this study is the detailed characterisation of Dp427, Dp140, and Dp71 expression across multiple brain regions, including isoform-specific in situ hybridisation with BaseScope. While previous research has generally identified the broader expression patterns of dystrophin isoforms in the CNS [14,37,43], our study provides a quantitative and region-specific profile of these isoforms in the mouse brain, particularly in the hippocampus, amygdala, cortex, and cerebellum.

In control C57BL/6J mice, our study revealed that Dp427c and Dp71 were the two isoforms with the highest transcript levels across all analysed brain regions, with some sub-regional differences. In regard to protein levels, Dp71 was the isoform with the highest expression. These results align with prior studies that have reported Dp71 as the predominant isoform in the CNS [14,15,44]. Additionally, its pronounced postnatal upregulation in regions like the cortex and cerebellum suggests a pivotal role in synaptic maturation and neural circuit refinement, possibly as well in angiogenesis, which could be linked to the more profound effect of Dp71 deficiency in the intellectual function of DMD patients [23]. These findings are supported by previous reports demonstrating Dp71’s localisation in perivascular astrocytes, forebrain glia, and postsynaptic compartments, particularly in inhibitory synapses [15,45].

Our study also provides evidence of distinct regional and isoform-specific expression patterns for Dp427. While Dp427c was predominantly expressed in cortical and forebrain regions, consistent with its role in GABAergic signalling [46], Dp427p1 and Dp427p2 were localised primarily to Purkinje neurons in the cerebellum. This finding is in agreement with previous studies showing Dp427p1/p2’s involvement in motor coordination and synaptic function within cerebellar circuitry [1,15]. Notably, we observed low levels of Dp427m, the muscle isoform, across all brain regions analysed, which aligns with observations of Dp427m expression in smooth muscle of murine brain arterioles [19] and in blood vessels of the human brain [16].

The BaseScope analysis further revealed sub-regional differences in dystrophin isoform transcript levels, with the hippocampus exhibiting the highest levels of all different *Dmd* transcripts in control mice. In contrast, regions such as the paracentral gyrus, corpus callosum, and substantia nigra displayed relatively lower expression. Dp71 and Dp427c were particularly abundant in the hippocampus and amygdala, regions critical for memory, emotion regulation, and fear responses [47,48,49]. A significant observation was the presence of Dp427c transcripts mainly in CA1-CA2, in contrast to Dp71, which was mainly present in the dentate gyrus. This remark could explain the differences observed between qPCR and BaseScope analysis, since in qPCR, the whole hippocampal area was analysed. These results are in contrast to human data, where Dp427c and Dp71 are expressed equally in the sub-areas of the hippocampus [16]. Although the mutation in *mdx^5cv^* mice results in the absence of Dp427, while Dp140 is also involved in *mdx52* mice, qPCR and BaseScope data still detect some *Dmd* mRNA expression of the respective missing isoforms. This can be attributed to the presence of short 5′ transcripts due to intronic sequence retention and alternative polyadenylation site [14] or the partial transcription of the *Dmd* gene before nonsense-mediated decay occurs [50], as well as the previously reported transcript imbalance observed for the *Dmd* mRNA [41]. It is important to highlight that when comparing C57BL/6J to mdx5cv and mdx52 models, a slight reduction, although not significant, in Dp71 isoform is observed in mdx52 animals. This finding is important for the CNS phenotypes observed in DMD patients, since further reduction in Dp71 levels likely further exacerbates the severity of behavioural and cognitive deficits, and the severity correlates with the isoforms lacking.

We did not observe any significant differences in protein levels of Dp427, Dp140, and Dp71 in C57BL/6J control mice across the distinct brain regions analysed. However, Dp71 levels are higher in all brain regions analysed, followed by Dp427 and Dp140. This could be attributed to the fact that Dp140 appears to be more highly expressed in embryonic and early postnatal stages and decreases in adulthood in the mouse [51] and human [15,16] brain. Due to the lack of sub-isoform-specific antibodies, we were not able to analyse protein levels of Dp427m and Dp427c. Of the two developmentally regulated isoforms of Dp427p—Dp427p1 and Dp427p2—only Dp427p1 is translated into a protein (GenBank: NM_004009 and NM_004010). However, due to the lack of a specific antibody, its protein levels cannot currently be measured. Dp427p2 transcript is not expected to result in protein production due to an additional 82 nucleotides directly after exon 1 that introduce a translational stop codon 24 nucleotides downstream of the same ATG codon included in the Dp427p1 transcript. Previous studies have not detected Dp427p2 protein in mouse tissue and cells [14], and Dp427p was virtually absent in human brain [15]. Thus, the Dp427 isoforms were pulled together in the protein analysis. It is also worth noting that the antibody epitope used on the WES analysis recognises epitopes located in exons 78–79, which are not present in Dp71 isoforms of the Dp71f family nor in Dp40 [7,8,9]. Thus, only Dp71d isoforms were detected in the WES experiment, but not Dp71f isoforms. These isoforms are differentially regulated during development and across brain structures, and the differences between RNA and protein analysis could thus be explained [52,53,54]. Moreover, the antibody did not detect Dp40, as it binds to the C-terminus. All the dystrophin isoforms, apart from Dp40, share the region that codes for a unique C-terminal sequence [9]. In contrast, it was detected in the transcript analysis, and a direct comparison for this dystrophin protein cannot be conducted. Another possibility for the differences between RNA and protein levels is dystrophin’s stability. The various sub-isoforms may be more stable and persist longer in certain brain regions, while others may be rapidly degraded. The degradation rates could depend on the activity of the proteasome or lysosome in certain brain areas [55]. Additionally, the partial transcription of the *Dmd* gene before nonsense-mediated decay occurs [50], and the previously reported transcript imbalance observed for the *Dmd* mRNA [41] could affect the translation process and produce different levels of dystrophins.

Another key finding of our study was the developmental regulation of Dp140 and Dp71 isoforms. Specifically, Dp140 expression was highest at P0, a critical stage of brain development associated with astrocytogenesis [56,57] and high levels at P7, an important time point for angiogenesis [58]. This is highly novel, as previous research has primarily focused on Dp140 expression in adults, with limited attention to its role in early development [14]. At the same time, Dp140 transcript has also been detected in neurons and astrocytes in the mouse [14]. We observed that Dp140 expression is still higher at P14 compared to P60 in the cortex and until P21 in the cerebellum, suggesting that this isoform may be especially important for synaptogenesis and glutamatergic neurotransmission during development, since these time points represent developmental landmarks of peak synaptogenesis at P14, including dendritic growth and spinogenesis at P21 [59,60,61,62]. The results of our study, along with human evidence [15,16], suggest a likely role of Dp140 during early development, particularly in regions such as the cerebellum and cortex, which are essential for motor coordination and cognitive function. The human study used unique transcriptomic data from the Allen Human Brain and BrainSpan atlases and determined that Dp140 exhibits very high expression during the early to mid-foetal stages in humans (week 8–10 post-conception), and this expression decreases significantly from the late foetal stages into adulthood in the human brain [15], similarly to our study. Notably, Dp140 is expressed in the cerebellum amongst all the brain regions in the adult human brain [15,16] with cell specific expression in Purkinje cells, granule cells, and dentate neurons of the cerebellum; dentate granule cells and hilar neurons of the hippocampus; and pyramidal neurons of the amygdala and neocortical areas [16] and continues to be postnatally produced in the mice, as observed in our study. The role of Dp140 postnatally is important since it has a significant role in glutamatergic neurotransmission [18]. Of interest, the behavioural phenotypic deficits of *mdx52* mice are improved postnatally after intra-cerebroventricular injection of antisense oligonucleotide drug-induced exon 53 skipping or intra-basolateral amygdala administration of a Dp140 mRNA-based drug [18].

In contrast to Dp140, our data showed that Dp71 maintained high expression in the adult brain, aligning with the findings in the human brain [15,16]. Previous studies have shown that Dp71 is expressed in both synapses and glial cells, such as astrocytes and Bergmann mouse glia, a result that emphasises the role of Dp71 in water and potassium homeostasis and synaptic function and plasticity [44]. It is worth noting that Dp71 levels at P60 in the cerebellum are higher compared to the cortex, which may be attributed to the late neuronal maturation observed in the cerebellum [63,64,65].

A limitation of the present study is that the isoform distribution was assessed at the cellular level, and cellular subtype-specific expression remains to be elucidated. Additionally, assessing the protein levels of specific dystrophin sub-isoforms was not feasible due to the unavailability of suitable antibodies, nor the developmental role of Dp427 due to very low expression in the present experimental conditions. Future studies may therefore focus on identifying and characterising the expression patterns of these specific proteins.

## 5. Conclusions

In conclusion, this study provides a comprehensive and region-specific analysis of dystrophin isoform expression in the C57BL/6J mouse brain, highlighting the distinct temporal and spatial dynamics of Dp427, Dp140, and Dp71. We identified Dp71 as the predominant isoform at the protein level and Dp427c and Dp71 at the RNA level. The differential expression patterns of Dp427 sub-isoforms, particularly Dp427c and Dp427p1/p2, suggest isoform-specific roles in cortical and cerebellar function. The BaseScope analysis further exhibited the high abundance of Dp71 and Dp427c in the hippocampus and amygdala, similarly to human brain studies. Additionally, the developmental regulation of Dp140 and its prominence during early postnatal stages, as observed in our study and in human studies, emphasise its role in synaptogenesis, neurotransmission, astrocytogenesis, and angiogenesis. While the lack of sub-isoform-specific protein data presents a limitation, our findings provide a foundation for future research into the functional roles of dystrophin isoforms in neurodevelopment and the neuropathophysiology of DMD.

## Figures and Tables

**Table 1:** Taqman probes designed to map dystrophin across the mouse brain.

Probe	Forward Primer 5′-3′	Reverse Primer 3′-5′	Probe	Location
Dp427m	GGACTGTTATGAAAGAGAAGATGTTCA	TGGCAGTTTTTGCCCTGTAAG	ACCTGCAGGATGGAAAACGCCTCC	Exon 1–2
Dp427c	GGCATGGAAGATGAAAGAGAAGA	GGCAGTTTTTGCCCTGTAAGG	ACCTGCAGGATGGAAAACGCCTCC	Exon 1–2
Dp427p1	CTTTCATCAGAAGAAACCTCAGACAT	CAGCCAAATGCTTTCCTATGAAG	AAATTCTGCGGAGGCTG	Exon 1–2
Dp427p2	CAGGCTTCCCTAAAGATGAAAGAG	GGCAGTTTTTGCCCTGTAAGG	ACCTGCAGGATGGAAAACGCCTCC	Exon 1–2
Dp140	GCTGACTGTTCTGAGCTAAAATCG	GCCATCCTGGAGTTCCTTAATAAG	ACCAGAAGGGGGTTTTG	Exon 1–2 of Dp140
Dp71	CACTGCCTGTGAAACCCTTACA	TGGGTCTCGTGGCCTTTG	CCATGAGGGAACACC	Exon 1–2 of Dp71
Dp40	AACGTGAGTAGTGGCAGAAGCA	TTTTGGCTGGGAGGAGTTCA	AGCAAACTTGCATTTGATA	Exon 9 of Dp40 absent on Dp71

## Data Availability

The data sets generated during and/or analysed during the current study are available in the Duchenne Data repository upon request, https://repository.duchennedatafoundation.org/ (accessed on 1 July 1996).

## Abbreviations

The following abbreviations are used in this manuscript:

ADHD	Attention-deficit/hyperactivity disorder
ASD	Autism spectrum disorder
CC	Coiled-coil domain
CNS	Central nervous system
CR	Cysteine rich domain
DMD	Duchenne muscular dystrophy
EF/ZZ	Helix-loop-helix
GABAA	γ-Aminobutyric acid type A
ISH	In situ hybridisation
IVC	Individually ventilated cages
mdx52	C57BL/6J mice lacking Dp427 and Dp140 isoform
mdx5cv	C57BL/6J mice lacking Dp427 isoform
NT	N-terminus
OCD	Obsessive-compulsive disorder
qPCR	Quantitative polymerase chain reaction
Ubc	Ubiquitin C
UTR	Untranslated region
WES	Western Blot capillary
WW	Double tryptophan
ZZ	Zinc-finger
